# A case of bilateral pneumothorax following computer‐tomography guided transthoracic biopsy in a woman with suspected pulmonary cancer

**DOI:** 10.1002/rcr2.1157

**Published:** 2023-07-17

**Authors:** Erik Sören Halvard Hansen, Meyya Bouazzi, Klaus Richter Larsen, Annemette Abield‐Nielsen, Oli Jacob Dalsgaard, Kasper Eibye

**Affiliations:** ^1^ Department of Respiratory Medicine Copenhagen University Hospital—Hvidovre Hvidovre Denmark; ^2^ Department of Respiratory Medicine Bispebjerg and Frederiksberg Hospital Copenhagen Denmark; ^3^ Department of Radiology Bispebjerg and Frederiksberg Hospital Copenhagen Denmark

**Keywords:** bilateral pneumothorax, respiratory failure, thymectomy

## Abstract

Computer‐tomography‐guided needle biopsies are useful for diagnosing, staging, and classification of peripheral pulmonary nodules. However, the procedure carries a risk of iatrogenic pneumothorax. This report describes a patient‐case where a woman had undergone a computer‐tomography guided biopsy. Approximately 4 hours following discharge the patient was admitted to the emergency ward with severe chest pain and dyspnea. Chest x‐ray revealed bilateral pneumothorax and subcutaneous emphysema at the biopsy site. Pleural drainage was administered on the patient's right side. Another chest x‐ray following drainage showed regression of pneumothorax on both sides thus indicating communicating pleural cavities. Medical history revealed that the patient had been thymectomized 2 years earlier and a computer tomography visualized that the patient lacked mediastinal separation of the two pleural cavities. It is possible that patients with a history of mediastinal or thoracic surgery should be observed longer following procedures carrying risk of iatrogenic pneumothorax.

## Graphical abstract

We present a case of delayed complication with bilateral pneumothorax (ptx) and subcutaneous emphysema at the biopsy site following computer‐tomography guided needle biopsy in a patient with a history of thymectomy. A history of thymectomy has previously been described as a cause of bilateral pneumothorax following procedures such as placement of right subclavian catheters however in such cases the onset of ptx was immediate. In our case, the patient had a normal chest x‐ray 2 hours following the biopsy and the onset started 4 h after the procedure. We suggest to increase the observation phase in such patients after an endo‐ or transthoracic intervention with a risk of pneumothorax.

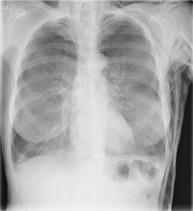

## INTRODUCTION

In patients with peripheral located pulmonary nodules suspicious of malignancy, computer‐tomography‐guided (CT‐guided) needle biopsy is often chosen for the purpose of staging and classification^1^ With this procedure follows a 30% risk of iatrogenic pneumothorax (ptx) which can be a dangerous condition if untreated.^2^, ^3^ However, in the daily clinic this type of biopsy is routinely performed and control chest x‐ray can be used to rule‐out iatrogenic ptx following the procedure.

## CASE REPORT

A 58‐year‐old woman, currently smoking with 40 pack‐years, under investigation for pulmonary cancer was brought into the emergency department at 5 PM with sudden onset of chest pain (Visual Analog Scale level 10) and dyspnea. Vital signs were normal except for a modest oxygen demand on 1 L/min to sustain a blood oxygenation of 95%. Electrocardiography revealed a sinusrhytm without suspicion of ischaemia or arrythmia.

**FIGURE 1:**
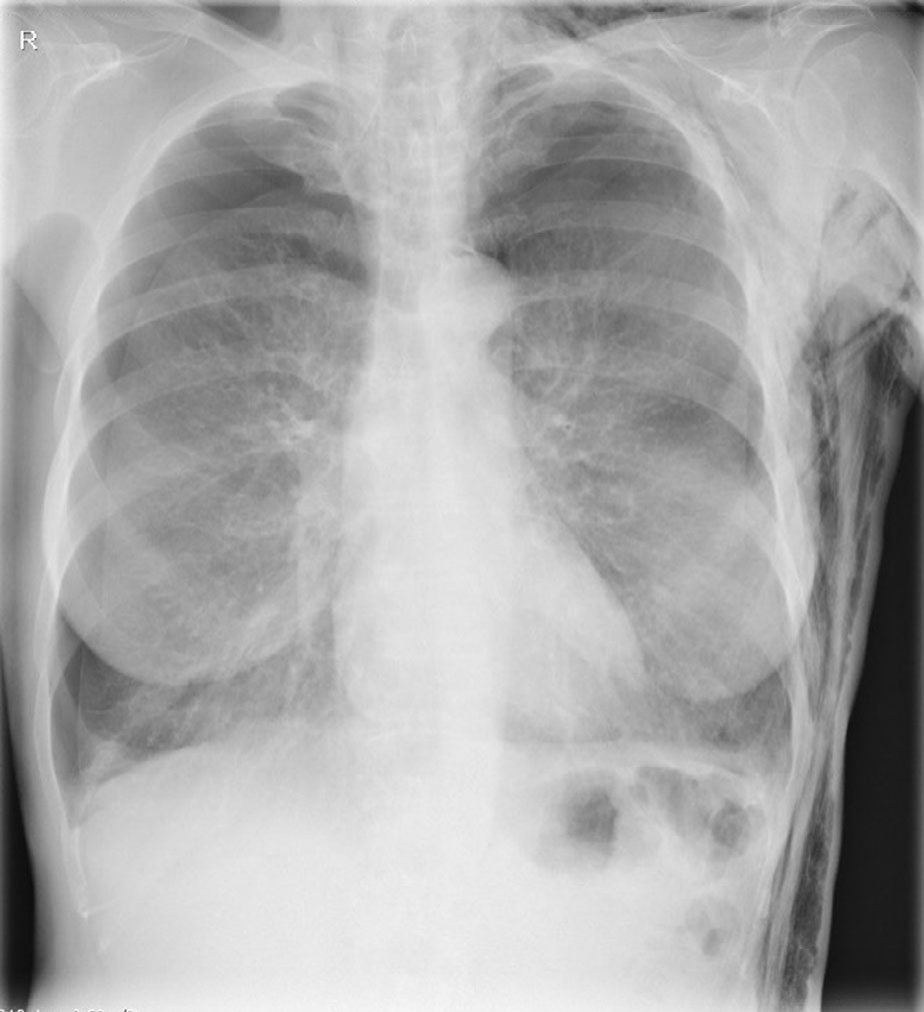
Chest x‐ray showing bilateral pneumothorax and subcutaneous emphysema before pleural drainage was administered.

**FIGURE 2:**
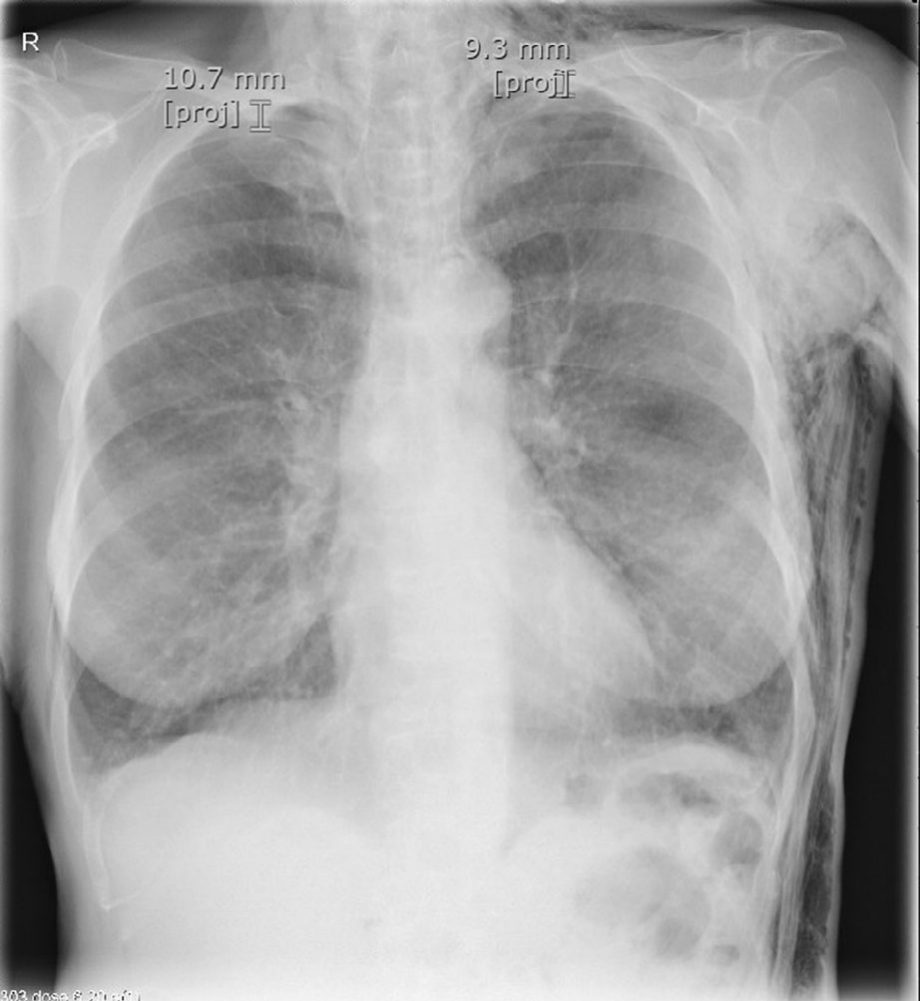
Chest x‐ray showing regression of both left and right pneumothorax following drain insertion on the patient's right side.

Earlier the same day at 10 AM the patient had undergone a CT‐guided needle biopsy of a peripheral pulmonary nodule located in the left lower lobe of the lung. A chest x‐ray 1 h following the procedure showed no sign of ptx or bleeding. In the emergency department the patient had a new chest x‐ray which revealed bilateral pneumothorax measuring >4 cm on both sides (Figure 1). Furthermore, on the left side, corresponding to the location of the transthoracic biopsy, a significant amount of subcutaneous emphysema was observed. A pigtail‐catheter French 12 was administered on her right side after evaluation by the treating clinician that this side had the best conditions for successful pleural drainage. Immediately following the procedure, the drain showed production of air and the patient experienced alleviation of respiratory symptoms. One hour later the drain was accidentally removed by the patient and a new chest x‐ray was performed which showed that the pneumothorax had completely resolved in the right side (Figure 2). Surprisingly, the left lung was also almost completely inflated indicating an effect of the right‐sided pigtail catheter on the left‐sided pneumothorax.

**FIGURE 3:**
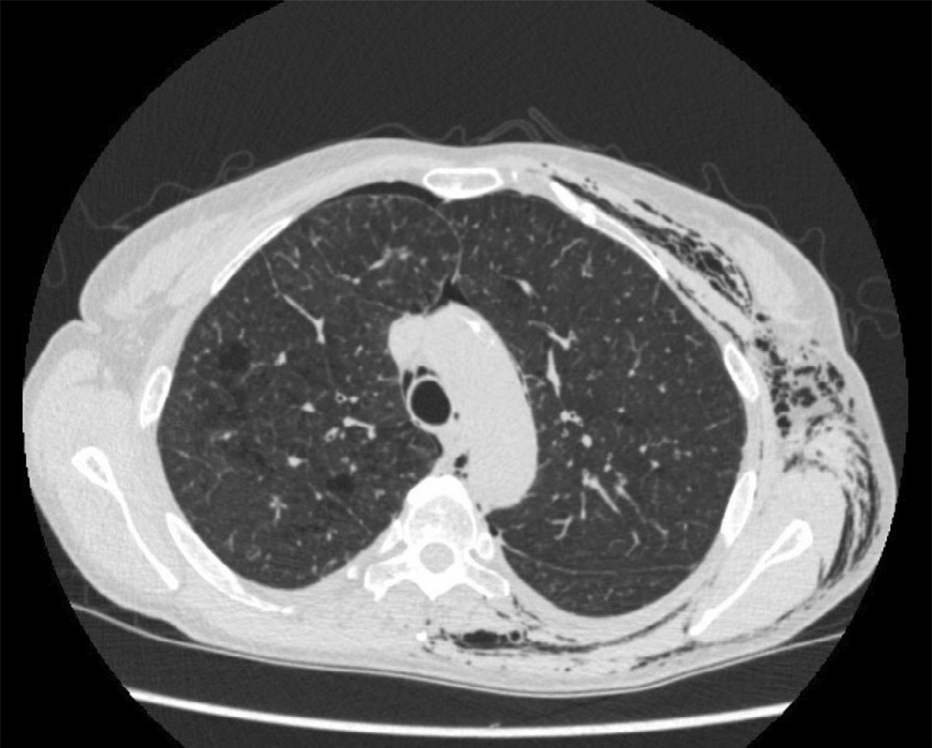
Computer tomography visualizing the patient's lack of mediastinal separation of the pleural cavities.

When asking more thoroughly into the woman's medical history this revealed that she 2 years prior had had a thymectomy. According to the patient's medical records, the thymectomy was without any complications and follow‐up computer tomography was without remarks concerning the thymectomy. Computer tomography the day following the acute onset of dyspnea and chest pain revealed that her pleural cavities were connected without normal mediastinal separation along with emphysema (Figure 3). The patient remained admitted for 8 days without further chest drains. Her bilateral pneumothorax almost completely regressed until discharge. Five days after discharge she met in the pulmonary outpatient clinic following a chest x‐ray which showed that her ptx was completely resolved.

The patient was diagnosed with a squamous cell carcinoma (T1bN0M0) and was successfully operated (video‐assisted thoracoscopic surgery segmentectomy) 3 weeks later without complications and with a planned oncological follow‐up.

## DISCUSSION

Ptx following CT‐guided transthoracic needle biopsies is a well‐known complication. Although the previous thymectomy seem to have led to connected pleural cavities it cannot be excluded that the reason for ptx was the patient's emphysema. However, the timing of the events (biopsy and bilateral ptx) indicate that the events are related. In this case, the bilateral ptx lead to acute respiratory failure warranting hospital admission and immediate treatment with a chest drain. Previous thymectomy has previously been described as cause of bilateral pneumothorax following procedures like placement of right subclavian catheters. However, previous case‐reports describe the onset of ptx as immediate.^4^ In our case, the patient had a normal chest x‐ray 2 hours following the biopsy. Therefore, to prevent serious adverse events, it is possible that patients with a history of thymectomy or other previous mediastinal surgery should be observed longer following endo‐ or transthoracic interventions with a risk of pneumothorax.

## AUTHOR CONTRIBUTIONS

Erik Sören Halvard Hansen and Kasper Eibye initiated the case report and developed the first manuscript. Klaus Richter Larsen, Meyya Bouazzi, Annemette Abield‐Nielsen, and Oli Jacob Dalsgaard provided intellectual feedback, comments, and corrections to the manuscript. All authors have read and approved the final version of the manuscript.

## CONFLICT OF INTEREST STATEMENT

None declared.

## ETHICS STATEMENT

The authors declare that appropriate written informed consent was obtained for the publication of this manuscript and accompanying images.

## Data Availability

Data sharing not applicable to this article as no datasets were generated or analysed during the current study.
